# Time-Sensitive Quality Metrics of Acute Stroke Care: A Comprehensive Stroke Center Experience

**DOI:** 10.5152/eurasianjmed.2024.24554

**Published:** 2024-10-01

**Authors:** Merve Korukcu, Alper Eren

**Affiliations:** 1 Department of Neurology, Ardahan State Hospital, Ardahan, Türkiye; 2 Department of Neurology, Ataturk University Faculty of Medicine, Erzurum, Türkiye

**Keywords:** Revascularization, stroke, stroke center, stroke onset, thrombolysis

## Abstract

**Background:**

The stroke center approach provides an effective solution for acute stroke management. Our study aims to systematically analyze our stroke center records to identify factors that affect acute stroke time-sensitive quality metrics.

**Methods:**

Data were prospectively collected from 524 acute stroke patients at the Comprehensive Stroke Center of Atatürk UniversityMedical Faculty Hospital between January 1, 2021, and September 30, 2021. Data collected included sociodemographic, clinical, admission type, initial National Institutes of Health Stroke Scale (NIHSS), treatment modality, and time-sensitive metrics and were statistically analyzed based on stroke quality metrics.

**Results:**

Patients with mild NIHSS scores (0-7) had longer onset-to-door (OTD) time, door-to-computed tomography (CT)/neurologist, and hospitalization times (*P* < .001). The OTD, door-to-CT/neurologist, and hospitalization times varied depending on the treatment methods used (*P* < .005). The OTD time was influenced by the patient’s level of education (*P* = .004), admission type (*P* < .001), and geographical location (*P* = .002). A moderate negative correlation was found between patients’ OTD time and baseline NIHSS (*r* = −0.270; *P* < 0.001).

**Conclusion:**

The results emphasize the significance of several factors, such as admission type, geographical location, and treatment methods, in shaping the time-sensitive quality metrics of stroke care. Additionally, the initial NIHSS score of patients plays a crucial role in both prehospital and in-hospital aspects of acute stroke management.

**Main Points**

- Since timely intervention is critical in acute stroke management, it is imperative to identify and address the factors impacting the acute stroke time metrics.
- Stroke severity has an impact on the time it takes for acute stroke patients to arrive at the hospital after their symptoms begin, and on the time until imaging and neurology consultation after arrival at the hospital.
- Education level, mode of admission to the hospital, focal symptoms/signs, and geographical disparities are among the factors that affect the time from the onset of patients’ symptoms to their admission to the hospital.

## Introduction

Despite significant advancements and strides in evidence-based acute care treatments, stroke remains a devastating ailment. The global incidence of stroke has increased by 70% over the past 3 decades, with a prevalence increase of 85% and a mortality increase of 43%.^1^ In 2019, stroke stood as the second most common cause of death globally, impacting 6.6 million people.^1^ Stroke-related disability-adjusted life years (DALYs) have increased by 32%, mainly due to population growth and aging.^2^

In this framework, the stroke community emphasizes the necessity for significant alterations in acute stroke management, including improvements in the utilization of tissue Plasminogen Activator (tPA), Endovascular Treatment (EVT), stroke center certification, neurocritical care, along with the adoption of mobile stroke units and telestroke.^3^ In 2005, the American Stroke Association Task Force outlined an organized approach to treating acute stroke patients.^4^ This led to certifications for various hospital levels, including Acute Stroke Ready Hospitals, Primary Stroke Centers (PSCs), Thrombectomy Capable Stroke Centers (TCSCs), and Comprehensive Stroke Centers (CSC).^5^ The metrics and data proposed for CSCs aim to monitor quality of care and support continuous improvement.^6^ This improvement is thought to be due in part to better adherence to quality criteria and guidelines for stroke care.^7^

As part of our study, we aimed to identify factors that may have an impact on stroke time-sensitive quality metrics. We conducted a systematic analysis of our stroke center-based registries containing stroke quality metrics.

## Material and Methods

Atatürk University Faculty of Medicine Hospital has been registered as a CSC by the Ministry of Health since 2021, providing 24/7 acute ischemic stroke treatment. The data were prospectively obtained from 524 acute stroke patients who provided consent, along with their relatives, between January 1, 2021, and September 30, 2021. The study excluded patients under 18 years of age and those diagnosed with hemorrhagic stroke. The study grouped patients based on their diagnosis of Cerebral Venous Thrombosis (CVT) and Transient Ischemic Attack (TIA) and arterial ischemic stroke using ICD-10.

### Study Design

The data collection form included sociodemographic details such as age, sex, educational level, and geographic location, as well as background information, including stroke-related risk factors, medications, and TOAST classification. Additionally, it recorded the mode of hospital admission (emergency health service, self-admission, or transfer from another hospital), drip-and-ship status, wake-up stroke, and focal neurological findings (motor, sensory, speech, visual, vestibular/vertigo, cognitive, and non-focal signs), as well as the FAST score. The severity of stroke upon admission was evaluated using the National Institutes of Health Stroke Scale (NIHSS) and stratified into NIHSS mild (0-6), moderate (7-15), or severe (≥16) based on the scores. At admission, all patients underwent at least one imaging modality (CT, magnetic resonance imaging (MRI), CTA, or MRA), assessed by 2 neurologists, with imaging times recorded on the data form. The treatments administered were classified as conservative treatment (such as LMWH, LMWH+antiagregan, acetylsalicylic acid, clopidogrel dual antiplatelet, and others), Intravenous recombinant tissue Plasminogen Activator (IV rt-PA), EVT, and a combination of IV rt-PA and EVT, with details recorded. The time from symptom onset to hospital admission (onset-to-door time) was calculated in minutes. Additionally, onset-to-door time was classified as 0-6 hours, >6 hours, and also as 0-4.5 hours, 4.5-6 hours, 6-24 hours, and >24 hours. The study measured the time intervals between hospital admission and various medical procedures, including CT/MRI imaging (door-to-CT/MRI), neurology consultation (door-to-neurologist), IV rt-PA and EVT administration (door-to-IV rt-PA and EVT), and hospitalization (door-to-hospitalization), all in minutes. The data was collected from various sources including patients, relatives, hospital automation systems, archives, and patient files completed by treating physicians.

The research protocol for this study received approval from the Atatürk University Faculty of Medicine Clinical Research Ethics Committee on June 24, 2021, with reference number 75. Informed consent was obtained from the patients who agreed to take part in the study. 

### Evaluation Process

Time-sensitive quality metrics were compared within baseline NIHSS groups and treatment modality groups. Additionally, sociodemographic factors (age, gender, patient education level, and geographic disparities) and prehospital factors (type of hospital admission and focal neurological findings) were compared between OTD time groups. Patients with TIA and CVT were excluded from statistical evaluation when analyzing time-sensitive quality metrics due to their different approaches to treatment and management compared to arterial ischemic stroke.

### Statistical Analysis

The study recorded patient demographic and clinical characteristics, diagnosis and treatment approaches, and discharge status data. Continuous variables were described using mean, median, SD, and range, while categorical variables were presented as counts and percentages. The normality of the data was evaluated using the Kolmogorov–Smirnov test, and the homogeneity of variances was assessed using the Levene test. The study utilized different statistical tests to compare variables between groups. The Kruskal–Wallis test was used for continuous variables with a non-normal distribution, such as most time-sensitive quality metrics related to NIHSS severity and treatment modalities, while 1-way ANOVA was applied to normally distributed variables with 3 or more groups, such as door-to-IV-rtPA time in relation to NIHSS severity. The Kruskal−Wallis test with Bonferroni correction was employed for variables that did not follow a normal distribution. The Pearson *χ*² test was employed to compare distributions between groups for categorical variables, such as the distribution of treatment modalities by NIHSS severity and the distribution of onset-to-door time groups by demographic and clinical characteristics. Spearman’s correlation coefficient was used to assess the relationships between onset-to-door time, NIHSS, and systolic blood pressure, while partial correlation analysis was employed to evaluate the relationship between NIHSS and onset-to-door time. Statistical significance was defined as a *P*-value below .05. Data analysis was conducted using SPSS version 27.0 (IBM SPSS Corp.; Armonk, NY, USA).

## Results

Of the 524 patients with acute stroke, 466 (88.9%) were diagnosed with ischemic stroke, 46 (8.8%) with TIA, and 12 (2.3%) with CVT.

### Demographic and Clinical Profiles of Patients

The study included 524 acute stroke patients with a mean age of 68.3 years (SD = 0.61), of which 54% were male. 43.5% of the patients had completed primary school. The majority of patients (54%) were transferred from other hospitals, and 51% of admissions were from rural areas. Although 63.5% of the patients had motor deficits, only 6.1% had visual impairment. At baseline, the NIHSS mean was 5.73 (SD 0.26) with a median of 4. Of the patients, 71.1% were in the mild group and 7.4% were in the severe group when grouped by NIHSS. Computed tomography scanning was performed on admission to 95.4% of patients, except for those who underwent IV rt-PA after a CT scan was performed at an external center and were referred to our hospital. Magnetic resonance imaging was performed on 88.4% of patients and CTA on 34.5%. Reperfusion treatments included IV rt-PA alone (5.3%), EVT alone (3.4%), and IV rt-PA+EVT (1.1%) (refer to Table 1).

The selection of acute stroke treatment was influenced by NIHSS severity (*P* < .001). In the NIHSS mild group, a higher prevalence of conservative treatment (77.2%) was observed, whereas IV rt-PA, EVT and IV rt-PA+EVT were used less frequently compared to the other groups (see Table 2).

### Evaluation of Time-Sensitive Quality Metrics

The door-to-EVT and door-to-IV rt-PA times did not exhibit significant differences across the NIHSS severity groups (*P* = .207 and *P* = .569, respectively). Yet, the NIHSS mild group exhibited longer onset-to-door (OTD), door-to-CT/neurologist/hospitalization times in contrast to the other groups (*P* < .001). Furthermore, the door-to-MRI time was found to be longer in the NIHSS mild group than in the moderate group (*P* = .007) (see Table 3).

The conservative treatment group exhibited a longer OTD time compared to the IV rt-PA and EVT groups (*P* < .001). Additionally, the conservative group had longer door-to-CT/MRI times than the IV rt-PA group (*P* < .001) and longer door-to-neurologist time (median 97 minutes) than all other groups (*P* < .001). Furthermore, the time from door-to-hospitalization was found to be longer in the conservative group compared to the IV rt-PA and IV rt-PA+EVT groups (*P* < .001) refer to Table 4.

There were no notable variances among the OTD time groups concerning gender or age groups. However, the OTD time was found to be dependent on the patient’s educational level (*P* = .004). For admissions exceeding 24 hours, the prevalence of illiteracy was higher than in the first 6 hours of admissions, and secondary school education was less common than in the 4.5-6 hours group. Additionally, the OTD time varied based on the mode of transmission (*P* < .001). In the group admitted within the first 4.5 hours, there were more admissions through Emergency Medical Services (EMS) compared to the group admitted after 6 hours. In the 6-24 hours group, transfers from another hospital were more common than in the first 4.5 hours group. Urban geography was more prevalent in the 0-4.5 hours group than in the group with admissions exceeding 6 hours, while rural geography was less common (*P* = .002). Motor deficits were more frequently detected in the 0-4.5 hours group compared to the other groups. Additionally, speech impairment was more common in this group than in the group with admissions exceeding 24 hours (*P* = .001 and *P* = .006, respectively). Patients admitted for more than 6 hours exhibited a greater prevalence of vestibular/vertigo symptoms in comparison to those admitted within the initial 4.5 hours (*P* < .001) (refer to Table 5).

There was a modest negative correlation between OTD time and NIHSS and systolic blood pressure (*r* = −0.270 and −0.110, respectively). Partial correlation analysis revealed a significant relationship only between NIHSS and OTD time (*r* = −0.175; *P* < .001), while there was no notable correlation observed between systolic blood pressure and onset-to-door (OTD) time (see Table 6).

## Discussion

The primary treatment modalities for acute ischemic stroke include IV thrombolysis and mechanical thrombectomy, and their efficacy is time-dependent. The aim of our study is to identify factors that may affect the time-sensitive metrics in stroke patients.

Concepts aimed at reducing prehospital delays to increase rates of acute revascularization have been studied extensively in recent years.^8-11^ The relationship between OTD time and NIHSS has been examined and supported in many previous studies.^12-15^ In this study, OTD time was found to be longer in the mild (≤6) NIHSS group than in the moderate and severe groups. Lyrer et al^16^ found that a higher NIHSS was associated with a shorter OTD interval. Lee et al^17^ showed that a higher initial NIHSS score had a significant negative correlation with late hospital arrival, similar to our study. This study also showed that systolic blood pressure was negatively correlated with OTD time; however, when partial correlation was applied, systolic blood pressure alone was not correlated. Clearly, severe symptoms are perceived as life-threatening and play a key role in the early hospitalization of acute stroke patients. Previous research has demonstrated an association between NIHSS and door-to-needle and door-to-imaging times.^18,19^ This study showed that when the NIHSS severity was mild, not only the OTD time but also the door-to-CT/neurologist/hospitalization time was prolonged, supporting that stroke severity is an important factor in the in-hospital management of acute stroke.

In addition to stroke severity, patient characteristics and prehospital management play a critical role in OTD time. Our study showed an association between OTD time and factors such as patient education level, mode of admission, focal symptoms/signs, and geographic disparities. In contrast to the findings of Derex et al^12^ who found no association between education level and delayed admission, our study was consistent with the findings of Lee et al^17^ who found that a lower education level was significantly associated with delayed hospital arrival compared with a higher education level. Similarly, our study observed a higher prevalence of illiteracy in admissions >24 hours compared to those within the first 6 hours. A high level of education increases the chances of recognizing symptoms and signs associated with acute ischemic stroke. This underscores that the education level is a valuable indicator of stroke awareness in society. Improved stroke awareness contributes to a shorter OTD time, thereby increasing the possibility of revascularization treatment for patients with acute ischemic stroke.

Many studies have emphasized the role of EMS in reducing OTD time.^20-26^ In addition, Chang et al^14^ observed a significant increase in prehospital delay with referral admissions. Consistent with the existing literature, our study found that EMS admissions were more frequent in the first 4.5-hour group than in the >6 hour group. In the 6-24-hour group, transfer from another hospital was more frequent than in the first 4.5-hour group. Our results also indicated that the patient’s living area influenced OTD time. In the 0-4.5-hour group, urban areas had a higher frequency compared to the >6 hour group, while rural areas had a lower frequency. Early admission plays a crucial role in the effectiveness of time-dependent treatments, and geographic factors may influence the opportunity for acute stroke treatment. Establishing a network of stroke centers is an effective strategy to address these limitations.

Wester et al^27^ showed an association between speech deficits and delay time. In contrast, our study showed that speech deficits were more frequent in the 0-4.5 hour group than in the >24 hour group. Our study also showed a higher prevalence of motor deficits in the 0-4.5 hour group compared to the other groups. In addition, vestibular/vertigo symptoms were more common in patients admitted >6 hours compared to those admitted within the first 4.5 hours. This supports the notion that patients perceive stroke symptoms primarily as weakness and speech impairment. Given the clinical spectrum of stroke, it is important to consider the societal education gap on this topic. Derex et al^12^ and Madsen et al^28^ showed that early presentation was significantly associated with female sex, whereas some studies^15,17,29^ have associated it with delayed presentation. Consistent with previous research^13,30-32^ our study found that gender did not have a significant effect on OTD time. While most studies^12,20,22,23,27^ did not identify age as a factor influencing prehospital delay, Chang et al^14^ observed an association between advanced age and delayed admission. Conversely, Lacy et al^13^ found an association between age ≥65 years and earlier admission. In our study, there was no notable discrepancy in terms of age and onset-to-door (OTD) time.

Several institution-wide stroke algorithms^35-37^ have focused on improving stroke outcome measures. In this context, time-sensitive metrics that are effective in both prehospital and in-hospital stroke management are emphasized. Our study revealed significant differences between treatment modalities and time metrics: OTD time was longer in the conservative treatment study group compared with the IV rt-PA and EVT groups, door-to-CT/MRI time was longer in the conservative study group compared with the IV rt-PA group, door-to-neurologist time was longer in the conservative study group compared with the other groups, and door-to-hospitalization time was longer in the conservative group compared with the IV rt-PA and IV rt-PA+EVT groups. Certainly, the patients who were not admitted within the acute revascularization treatment window and thus underwent conservative treatment are expected to have a longer OTD time. However, our study showed that in-hospital treatment of patients scheduled for revascularization was more rapid. These findings support the stroke center approach of taking steps to achieve targeted quality time metrics for acute stroke.

Our study is subject to several limitations that should be acknowledged. First, our data are limited to patients admitted to our hospital and may not reflect national data. Another limitation is the absence of more comprehensive data regarding economic standing and individual behaviors, limiting the exploration of additional hypotheses related to the acute stroke process. The third and most important limitation of our study is the overlap with the period of the Covid-19 pandemic. This situation affected the number of hospital admissions, the processes of hospitalization, and in-hospital procedures. In addition, during this period, difficulties in nationwide transport for thrombolytic treatment affected the treatment data in our study.

As acute stroke treatments are time-sensitive, intervening during both pre-hospital and in-hospital periods presents an opportunity to improve treatment outcomes. Identifying and addressing factors that affect acute stroke time metrics is crucial for successful acute stroke management. In this context, it is important to emphasize training and related projects that can increase awareness of stroke symptoms and the use of emergency services. Our study supports the idea that such efforts can have an impact on onset-to-door time. Additionally, expanding acute stroke units can help improve treatment options for patients in rural areas and reduce the risk of delayed admission to the hospital.

## Data Availability Statement

The data that support the findings of this study are available on request from the corresponding author.

## Figures and Tables

**Figure 1.**
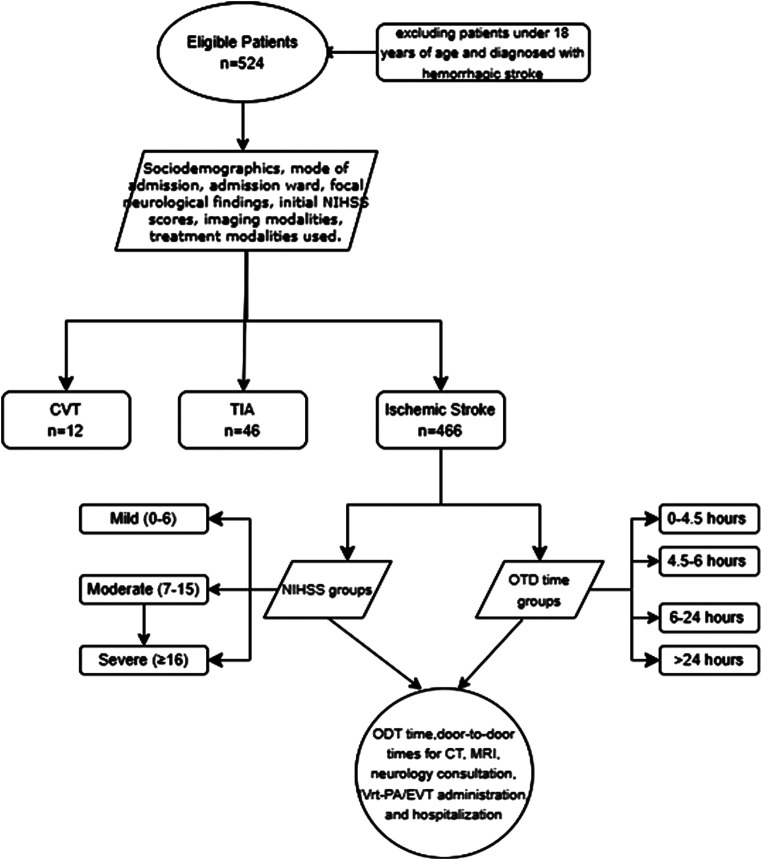
Flowchart of patients included in the study.

**Table 1.** Demographics and Clinical Characteristics

	Acute Ischemic Stroke (N = 524)		
	N (%) or Mean (SD)		N (%) or Mean (SD)
Age	68.3 (0.61)	Focal neurological signs	
Sex		Motor	333 (63.5)
Female	241 (46)	Sensory	22 (4.2)
Male	283 (54)	Speech	209 (39.9)
Patient education level		Visual	32 (6.1)
Not literate	179 (34.2)	Vestibular/vertigo	57 (10.9)
Primary school	228 (43.5)	Cognitive	68 (13)
Secondary school	49 (9.4)	Non-focal sign	28 (5.3)
High school	49 (9.4)	Treatment protocol	
University	19 (3.6)	*Conservative*	472 (90.1)
Geographic disparities		LMWH	84 (16)
Urban	230 (43.9)	LMWH+antiagregan	312 (59.5)
Rural	267 (51)	Acetylsalicylic acid	8 (1.5)
Remote urban/rural area	27 (5.2)	Clopidogrel	5 (1)
Mode of admission		Dual antiplatelet	62 (11.8)
Through emergency health service	70 (13.4)	Others	2 (0.4)
Self-admission	171 (32.6)	*IV rt-PA*	28 (5.3)
Shipping from another hospital	283 (54)	*EVT (include IV rt-PA+EVT)*	18 + 6 (3.4 + 1.1)
Imaging radiology at admission		Mechanical thrombectomy	6 (1.1)
CT	500 (95.4)	IA rt-PA	9 (1.7)
MRI	463 (88.4)	Thrombectomy+IA rt-PA	9 (1.7)
CTA	181 (34.5)	*IV rt-PA+EVT*	6 (1.1)
MRA	81 (15.5)	Initial NIHSS	5.73 (0.26)

CT, computed tomography; CTA, computerized tomography angiography; ECASSII, European Cooperative Acute Stroke Study; EVT, endovascular treatment; HI, hemorrhagic Infarction; IA, intra arterial; IV rt-PA, intravenous recombinant tissue plasminogen activator; LMWH, low-molecular-weight heparin; MRI, magnetic resonance imaging, MRA, magnetic resonance angiography; mRS, modified Rankin Scale; NIHSS, National Institutes of Health Stroke Scale; PH, parenchymal hemorrhage.

**Table 2.** Distribution of Ttreatment Modality on NIHSS Severity

		NIHSS Groups*				*df*	P**
		Mild	Moderate	Severe	Total		
Treatment modalities, n (%)	Conservative	328 (77.2)^a^	76 (17.9)^b^	21 (4.9)^c^	425 (89.1)	6	**<.001**
	IV rtPA	10 (35.7)^a^	12 (42.9)^b^	6 (21.4)^b^	28 (5.9)		
	EVT	1 (5.6)^a^	9 (50)^b^	8 (44.4)^b^	18 (3.8)		
	IVrtPA+EVT	-^a^	2 (33.3)^b^	4 (66.7)^b^	6 (1.3)		

*NIHSS groups: mild: 0-6, moderate: 7-15, severe: ≥16.

**Chi-Square test.

^a-c^shows differences in treatment modalities.

**Table 3.** Time-Sensitive Quality Metrics on NIHSS Severity

Time Metrics (minutes)	NIHSS Groups			*df*	P*
	Mild	Moderate	Severe		
Onset-to-door time	574 (28-21600)^a^	297 (25-5760)^b^	172 (4-900)^b^	40.906	**<.001**
Door-to-CT time	32 (5-507)^a^	21 (4-432)^b^	20.5 (5-61)^b^	39.04	**<.001**
Door-to-MRI time	93 (8-872)^a^	80 (8-439)^b^	75.5 (19-226)^ab^	9.836	**.007**
Door-to-neurologist time	120 (0-946)^a^	60 (0-335)^b^	39 (1-170)^b^	52.959	**<.001**
Door-to-hospitalization time	171 (0-1440)^a^	153 (1-595)^b^	99 (1-311)^b^	30.898	**<.001**
Door-to-EVT time	280 (280-280)	54 (29-261)	48 (8-210)	3.147	.207
					P**
Door-to-IV-rtPA time	98.22 (11.8)	103.83 (14.84)	80.28 (19.04)		.569

^a-b^There is no difference between groups with the same letter for each metric (Bonferroni test).

*Kruskal–Wallis, median (min-max).

**One-way ANOVA, mean (SD).

**Table 4.** Time-Sensitive Quality Metrics for Treatment Modalities

Time Metrics (min)	Treatment Modalities				*χ*2	P*
	Conservative	IV rt-PA	EVT	IV rt-PA+EVT		
Onset-to-door time	465 (13-21 600)^b^	69 (25-165)^a^	106 (29-321)^a^	188 (4-352)^ab^	78.428	**<.001**
Door-to-CT time	29 (3-813)^b^	17 (5-176)^a^	19 (10-250)^ab^	13 (8-43)^ab^	18.588	**<.001**
Door-to-MRI time	91.5 (8-872)^b^	52.5 (28-124)^a^	89 (11-255)^ab^	19 (19-19)^ab^	12.218	**.007**
Door-to-neurologist time	97 (0-946)^b^	42 (12-120)^a^	36.5 (1-224)^a^	18 (12-170)^a^	40.356	**<.001**
Door-to-hospitalization time	166 (0-1440)^b^	97 (1-221)^a^	107.5 (1-348)^ab^	52.5 (20-238)^a^	39.503	**<.001**

CT, computerized tomography; EVT, endovascular treatment; IV rt-PA, intravenous recombinant tissue plasminogen activator; MRI, magnetic resonance imaging.

^a-b^There is no difference between groups with the same letter for each metric (Bonferroni Test),

*Kruskal–Wallis, median (min-max).

**Table 5.** Distribution of Onset-to-Door Time Groups by Demographic and Clinical Characteristics

		Onset-to-Door Time Groups						
		0-4.5 Hours	4.5-6 Hours	6-24 Hours	>24 Hours	Total	*χ* ^2^	P*
Sex	Female	67 (40.1)	22 (43.1)	78 (47.0)	46 (56.1)	213 (45.7)	5.914	.116
	Male	100 (59.9)	29 (56.9)	88 (53.0)	36 (43.9)	253 (54.3)		
Patient education level	Not literate	46 (27.5)^a^	12 (23.5)^a^	59 (35.5)^ab^	41 (50.0)^b^	158 (33.9)	29.231	**.004**
	Primary school	80 (47.9)^a^	22 (43.1)^a^	70 (42.2)^a^	32 (39.0)^a^	204 (43.8)		
	Secondary school	17 (10.2)^ab^	8 (15.7)^b^	16 (9.6)^ab^	1 (1.2)^a^	42 (9)		
	High school	21 (12.6)^a^	7 (13.7)^a^	11 (6.6)^a^	7 (8.5)^a^	46 (9.9)		
	University	3 (1.8)^a^	2 (3.9)^a^	10 (6.0)^a^	1 (1.2)^a^	16 (3.4)		
Mode of admission	Through EMS	38 (22.8)^a^	7 (13.7)^ab^	12 (7.2)^b^	5 (6.1)^b^	62 (13.3)	26.225	**<.001**
	Self-admission	61 (36.5)^a^	18 (35.3)^a^	54 (32.5)^a^	30 (36.6)^a^	163 (35)		
	Shipping	68 (40.7)^a^	26 (51.0)^ab^	100 (60.2)^b^	47 (57.3)^ab^	241 (51.7)		
Geographic disparities	Urban	96 (57.5)^a^	25 (49.0)^ab^	64 (38.6)^b^	26 (31.7)^b^	211 (45.3)	20.697	**.002**
	Rural	67 (40.1)^a^	24 (47.1)^ab^	92 (55.4)^b^	50 (61.0)^b^	233 (50)		
	Remote urban/rural	4 (2.4)^a^	2 (3.9)^a^	10 (6.0)^a^	6 (7.3)^a^	22 (4.7)		
Age groups	<55	34 (20.4)	9 (17.6)	26 (15.7)	15 (18.3)	84 (18)	1.252	.741
	>55	133 (79.6)	42 (82.4)	140 (84.3)	67 (81.7)	382 (82)		
Focal signs/ symptoms	Motor	123 (73.7)^a^	27 (52.9)^b^	93 (56.0)^b^	45 (54.9)^b^	288 (61.8)	15.644	**.001**
	Sensory	4 (2.4)	4 (7.8)	10 (6.0)	2 (2.4)	20 (4.3)	4.926	.177
	Speech	80 (47.9)^a^	19 (37.3)^ab^	60 (36.1)^ab^	21 (25.6)^b^	180 (38.6)	12.396	**.006**
	Visual	10 (6.0)	3 (5.9)	12 (7.2)	7 (8.5)	32 (6.9)	0.67	.88
	Vestibular/ vertigo	7 (4.2)^a^	7 (13.7)^ab^	21 (12.7)^b^	18 (22.0)^b^	53 (11.4)	18.196	**<.001**
	Cognitive	23 (13.8)	4 (7.8)	14 (8.4)	14 (17.1)	55 (11.8)	5.389	.145
	Non-focal	5 (3.0)	6 (11.8)	9 (5.4)	6 (7.3)	26 (5.6)	6.3	.098

EMS, emergency medical services.

^a-b^There is no difference between groups with the same letter.

*Chi-square test, N (%).

**Table 6.** Relationship Between Onset-to-Door Time and NIHSS, Systolic Blood Pressure

	Onset-to-Door Time			
	r_1_	*P*	r_2_	*P*
NIHSS	−0.27	**<.001**	-0.175	**<.001**
Systolic blood pressure	−0.11	**.018**	-0.048	.329

NIHSS, National Institutes of Health Stroke Scale.

r_1_, Spearman correlation.

*r*_2_, partial correlation.
